# Just regionalisation: rehabilitating care for people with disabilities and chronic illnesses

**DOI:** 10.1186/1472-6939-7-9

**Published:** 2006-08-29

**Authors:** Barbara Secker, Maya J Goldenberg, Barbara E Gibson, Frank Wagner, Bob Parke, Jonathan Breslin, Alison Thompson, Jonathan R Lear, Peter A Singer

**Affiliations:** 1 Joint Centre for Bioethics, University of Toronto, 88 College Street, Toronto, Ontario, Canada; 2 Toronto Rehabilitation Institute, 550 University Avenue, Toronto, Ontario, Canada; 3 Department of Philosophy, Michigan State University, 503 South Kedzie Hall, East Lansing, Michigan, USA; 4 Department of Physical Therapy, University of Toronto, 500 University Avenue, Toronto, Ontario, Canada; 5 Toronto Community Care Access Centre, 250 Dundas Street West, Suite 305, Toronto, Ontario, Canada; 6 Humber River Regional Hospital, 2111 Finch Avenue West, North York, Ontario, Canada; 7 North York General Hospital, 4001 Leslie Street, North York, Ontario, Canada; 8 Centre for Research on Inner City Health, St. Michael's Hospital, 70 Richmond Street East, 4^th ^Floor, Toronto, Ontario, Canada

## Abstract

**Background:**

Regionalised models of health care delivery have important implications for people with disabilities and chronic illnesses yet the ethical issues surrounding disability and regionalisation have not yet been explored. Although there is ethics-related research into disability and chronic illness, studies of regionalisation experiences, and research directed at improving health systems for these patient populations, to our knowledge these streams of research have not been brought together. Using the Canadian province of Ontario as a case study, we address this gap by examining the ethics of regionalisation and the implications for people with disabilities and chronic illnesses. The critical success factors we provide have broad applicability for guiding and/or evaluating new and existing regionalised health care strategies.

**Discussion:**

Ontario is in the process of implementing fourteen Local Health Integration Networks (LHINs). The implementation of the LHINs provides a rare opportunity to address systematically the unmet diverse care needs of people with disabilities and chronic illnesses. The core of this paper provides a series of composite case vignettes illustrating integration opportunities relevant to these populations, namely: (i) rehabilitation and services for people with disabilities; (ii) chronic illness and cancer care; (iii) senior's health; (iv) community support services; (v) children's health; (vi) health promotion; and (vii) mental health and addiction services. For each vignette, we interpret the governing principles developed by the LHINs – equitable access based on patient need, preserving patient choice, responsiveness to local population health needs, shared accountability and patient-centred care – and describe how they apply. We then offer critical success factors to guide the LHINs in upholding these principles in response to the needs of people with disabilities and chronic illnesses.

**Summary:**

This paper aims to bridge an important gap in the literature by examining the ethics of a new regionalisation strategy with a focus on the implications for people with disabilities and chronic illnesses across multiple sites of care. While Ontario is used as a case study to contextualize our discussion, the issues we identify, the ethical principles we apply, and the critical success factors we provide have broader applicability for guiding and evaluating the development of – or revisions to – a regionalised health care strategy.

## Background

Regionalisation's promise of more effectively integrating health and social services is especially important for people with disabilities and chronic illnesses transitioning to and/or living in the community. Without effective service integration, people with disabilities and chronic illnesses may be forced to live in institutional settings in order to access health care, and those who live in the community may either live at risk or be highly dependent on family members [1].

Furthermore, fragmentation of services can result in persons becoming lost in a complex system of bureaucracy, multiple caregivers, and diverse funding mechanisms resulting in potential gaps in service and increasing caregiver burden [2].

In this paper, we consider regionalisation from the perspective of disability ethics. Disability ethics integrates disability studies and normative ethics, including bioethics, where the health care experiences of people with disabilities are concerned [3,4]. While "disability ethics" is a broad field that addresses a range of issues using various approaches and perspectives, this field is marked by a basic concern with examining the ethical implications of prevailing social, cultural and political arrangements for people with disabilities and chronic illnesses and for society as a whole [5]. Within health care, the primary goals for people with disabilities and chronic illnesses include improving function, enabling participation, and enhancing quality of life. These priorities are often perceived to be less urgent and less compelling than the "life-and-death" issues that arise in acute health care settings, however, and thus the ethical issues relating to disability and chronic illnesses are under-researched in bioethics [5-8]. These ethical issues are plentiful, however, and increasingly pressing given rising rates of disability (secondary to an increasing – and aging – population, technological advances that preserve and prolong life, increasing life expectancy, and a rise in chronic illnesses) and given a growing understanding of accessibility and participation in society as basic human rights [2]. Disability ethics often adopts a social justice framework [9,10] in its examination of issues of marginalization as they relate to the health and quality of life for people with disabilities and chronic illnesses. The core assumptions of disability ethics are that (a) people with disabilities are marginalized as a group, the interests of which are routinely subordinated to those of able-bodied people and (b) such marginalization is morally and politically unjust and must be addressed.

Although there is ethics-related research into disability and chronic illness [10-14] and studies of regionalisation experiences [15,16] (including the impacts of regionalisation on the continuum of care [17] and resource allocation [18]), and research directed at improving health systems for these patient populations [18], to our knowledge there have been no investigations into the ethics of regionalisation, particularly with a focus on the implications for persons with disabilities and chronic illness. This paper aims to examine the ethics of regionalisation for people with disabilities and chronic illnesses using the newly developed regionalization strategy in Ontario, Canada as a case study. While Ontario is used to contextualize our discussion, the issues we identify, the ethical principles we apply, and the critical success factors we provide have broader applicability for guiding and evaluating the development of – or revisions to – a regionalised health care strategy.

## Discussion

Ontario has served as a "control case" in the Canadian regionalisation literature over the past decade because it was the only Canadian province that did not have formal regional health authorities in place. This is about to change. Ontario is on the brink of implementing regionalised health care in the form of fourteen Local Health Integration Networks (or LHINs) [19]. The Ontario LHINs model is being promoted as a means for transforming the province's health care system "from a collection of services to a true health care system." [20] The Ministry of Health and Long Term Care's (MOHLTC) vision is that the LHINs will help improve patient-centredness, responsiveness to local health needs, and "local capacity to plan, coordinate, integrate, and fund the delivery of health services at the community level." [20] The structure of the LHINs is meant to enable better health care service integration and "ease the movement of people across the continuum of care so that they get the best care, in the most appropriate setting, when they need it." [20]

The move toward LHINs provides a rare opportunity to address systematically the unmet needs of people with disabilities and chronic illnesses requiring in-patient, ambulatory, and community-based rehabilitation services in Ontario, as well as other support services to improve function, enable participation in the community, and enhance quality of life. The potential for the LHINs structure to deliver patient-centred care for people with disabilities and chronic illnesses was recognised by participants in a series of community workshops conducted by the MOHLTC in January 2005 to establish priorities for LHINs [21]. When asked to identify the key integration issues faced in health care delivery, the approximately four thousand participants (including representatives of patient advocacy and community groups, the public, health care providers, provider organizations, health-related associations, and the MOHLTC) frequently cited services for people with disabilities, chronic illness management, community support services, and rehabilitation [21]. The participants stressed the need for integrated services and a true continuum of care in the new health care delivery model, specifically calling for "vertical strategies linking different levels of care or parts of the care continuum, i.e. acute, rehab, complex continuing care, and long term care" and "horizontal strategies linking providers at similar levels of care, i.e. rehab networks, cardiac networks." [21]

In addition to soliciting community input in identifying key integration opportunities, the MOHLTC published the following governing principles for the LHINs [20]:

1. Equitable access based on patient need

2. Preserves patients' choice

3. Measurable, results-driven outcomes based on strategic policy formulation, business planning and information management

4. People-centred, community-focused care that responds to local population health needs

5. Shared accountability between providers, government, community and citizens

These principles are recognisably ethical in nature insofar as they identify normative ethical values. They provide very limited ethical guidance, however, as they were not fully articulated or defined in the LHINs documents. Our aim was to flesh out these established principles in order to provide guidance on how they apply to commonly encountered issues and situations affecting persons with disabilities and/or chronic illnesses.

## Methods

The LHINs principles were identified based on a MOHLTC-organized group consensus process that did not define or describe the principles, or articulate an overarching ethical framework and/or set of normative ethical assumptions. Nor was there any reference in the LHINs documents to how the principles would be applied in situations of competing claims for limited resources. Despite these limitations, we recognized the value of working with the LHINs published principles and thus we undertook to interpret the principles based on a conceptualization of social justice consistent with Iris Marion Young's work in *Justice and the Politics of Difference *[22] and its application in disability ethics [23]. Young's approach goes beyond distributive issues to address the marginalization of excluded groups by affirming social group differences in public policy rather than treating justice as primarily a matter of distributing material resources among persons assumed to be roughly equal in social status and power.

Drawing from this social justice framework, we examined the principles in relation to series of composite vignettes. We developed the vignettes based on the ethical issues ranked most important by 21 key stakeholders at three Toronto rehabilitation and community care centres (Toronto Rehabilitation Institute, Bloorview Kids Rehab and Toronto Community Care Access Centre), and by the community stakeholder groups organized by the MOHLTC to help identify "key integration opportunities" for the LHINs. In our analyses, we used a process of wide reflective equilibrium as articulated by Daniels [24]. This involved moving back and forth from our considered moral judgments about the vignettes to the LHINs principles to our social justice lens, and refining and revising these until we reached an "equilibrium" or a coherence among our ethical judgments, principles and background theories. The "critical success factors" that follow the case vignettes below were derived from these analyses in an effort to provide concrete guidance to policy makers and administrators (in this case the LHINs leadership) in honouring these principles.

In this paper, we did not address directly the issue of how to resolve competing claims with limited resources but rather viewed our analysis as a necessary first step towards ensuring that those with disabilities and chronic illnesses are included in priority setting discussions. We do so by demonstrating how the principles articulated by the LHINs (which are more or less common amongst public health care organizations) have direct implications for the health and welfare of these populations. Once this is understood, the obvious next steps include consideration of ethical frameworks for resource allocation and priority setting.

### Analysis

In Table 1, we offer an interpretation of the LHINs principles in the context of disability ethics to guide our analysis and advice. Note that in working with the MOHLTC's five-principle formulation, we have made minor modifications to how the principles are named and organized as part of the work of fleshing out the principles for the context of persons with disabilities and chronic illnesses. Our principles 1, 2 and 4 mirror the language of the MOHLTC. We have, however, rolled the MOHLTC's original principle #3 ("Measurable, results-driven outcomes based on strategic policy formulation, business planning and information management") into the principle of shared accountability. In addition, we have separated the MOHLTC's original principle #4 ("People-centred, community-focused care that responds to local population health needs") into two distinct yet related principles – our principles #3 and #5. Finally, we have opted to use the more common terminology (reflected in other MOHLTC documents) of "patient-centre care" rather than "people-centred care."

**Table 1:** Guiding principles of LHINs

**Guiding principle**	**Interpretation**
**1. Equitable access based on patient need**	*Equitable access based on patient need *is defined in MOHLTC documents as "access to appropriate quality care when (patients) need it." [25] Equitable or fair access requires sufficient resources to offer these services and to eliminate social barriers that may keep patients from accessing those services. Because patient need is often best defined by the actual patients, community members, including those with chronic conditions and disabilities, must participate in setting goals and organizing community health programs.
**2. Preserving patient choice**	*Preserving patient choice *is discussed in the MOHLTC documents as permitting persons to seek treatment outside of their LHIN [25]. We read this important principle more broadly and suggest that it ought to mean that patients or their substitute decision makers (SDMs) are offered a meaningful range of options to consider when making decisions about health services along the continuum of care. This includes, but is not limited to, accessing services outside of the patient's regional health district.
**3. Responsiveness to Local Population Health Needs**	To be responsive to the health needs of a local population means being attuned to the specific health problems within the community and being able to address those needs with effective targeted programs and services. Because communities are unique, complex, and dynamic, there must be broad community representation in the organisation of services.
**4. Shared accountability**	The concept of *shared accountability*, as advanced by the MOHLTC, is a bilateral initiative between the province and health care delivery agencies to increase responsibility and transparency with respect to priority setting and measuring. Shared accountability requires a shared commitment to, and responsibility for, improving health care delivery between governments, LHINs and health care providers. This includes clear delineations of responsibility and the alignment of authority and accountability. Specifically, "the government will do its job of leading and managing the health care system instead of micro-managing, and regional structures will likewise be accountable for planning and coordinating care, cutting across the current silos of both programs and funding, and be responsible for the day-to-day delivery of care on behalf of the individual patients and populations they serve." [26]
**5. Patient-centred care**	*Patient-centred care *embraces a philosophy of respect for and partnership with people receiving services by building processes into the health system that recognize patients' perspectives in identifying care needs and ensuring that services are accessible and fit for the context in which persons live [27,28].

In what follows, we explore how these principles apply to "integration opportunities" relevant to people with disabilities and chronic illnesses, namely: (i) rehabilitation and services for people with disabilities; (ii) chronic illness and cancer care; (iii) senior's health; (iv) community support services; (v) children's health; (vi) health promotion; and (vii) mental health and addiction services. In a series of composite case vignettes that introduces challenging examples specific to each integration topic, we describe how the principles in Table 1 apply. We then provide critical success factors derived from the analysis to guide the LHINs in upholding the MOHLTC-identified principles in responding to the needs of people with disabilities and chronic illnesses.

#### Rehabilitation and services for people with disabilities: the need for affordable and accessible accommodations

*Jean-Marc Dublas is a 30 year-old man with a C5/6 incomplete quadriplegia acquired while diving. He is an in-patient ready to be discharged from a spinal cord rehab facility. His length of stay (LOS) is far longer than the target LOS because there is no appropriate place for Jean-Marc to be discharged to. Given that it is not financially feasible for him to adapt his pre-injury home, he wants to live independently in a Support Service Living Unit (SSLU) in the community where he would receive appropriate attendant care services; however none is available and the projected wait for such accommodation is 2–3 years. The unit manager and social workers have explored all options and the only place to discharge Jean-Marc is to a complex continuing care facility where patients are on average much older and less functional than Jean-Marc. Jean-Marc is adamantly refusing to be discharged to this facility. Staff, while sympathetic, maintain that Jean-Marc cannot stay in the rehab facility much longer. Patients with similar injuries who are injured while working or in motor vehicle accidents are eligible to be discharged into the community sooner because the Workplace Safety and Insurance Board (WSIB) or motor vehicle insurance can fund home-care and personal support workers or provide financial support for individuals to privately purchase needed supportive care*.

#### LHINs principles

While all five of the LHINs guiding principles are relevant to this difficult case involving discharge planning and placement, the principle of *equitable access based on patient need *appears most salient. People with disabilities and chronic illnesses often perceive rehabilitation and social services (related, for example, to education, employment, and community living) to be equally important as acute medical needs [29,30]. However, acute health care services receive priority funding before important community and social services, such as accessible housing and attendant care services. If we take seriously the principle of *equitable access based on patient need*, the first step would be to determine in consultation with key stakeholder groups what people with disabilities and chronic illnesses *need *– that is, to identify a set of core programs and services, including high priority services. The principles of *preserving patient choice, responsiveness to local population needs*, and *patient-centred care *reinforce the above direction. Discharging patients against their will to the sole "option" of an institutional setting would seem inconsistent with these principles, particularly when in-patient care costs are greater than standard home-care costs for patients with disabilities and chronic illnesses, even those with quadriplegia [31,32].

Once core programs and services are identified, the *access *component of this principle would require reducing the gap between supply and demand, likely facilitated in part by the reinvestment of funds from institutional to community sectors. Reducing waiting times for housing, community and social services, along the lines of the Wait Times Strategy in relation to health services [33], would also improve access.

*Equitable *access, however, would require more than an increase or reallocation of resources and reduction in average wait times. It would also require better integration and coordination of these resources within and across the relevant Ministries such that a consistent range of core programs and services be made available across communities. *Equitable access based on patient need *(as opposed to the source of the injury, or where and how the injury was sustained) would mean that core programs and services would be available regardless of the type of payer; thus, a publicly funded range of services would be available when insurance coverage is not – a range of publicly funded programs and services programs at least approximating the range offered by insurers. In addition to developing a transparent system of prioritizing persons waiting for services, equity would require wait times for publicly funded services be comparable to those for insured and private services.

#### Critical success factors

Honouring these principles thus requires attention to *integrating services *across health and social service ministries and departments to identify and address the housing and care needs of person with disabilities and chronic illnesses while reducing financial burden to facilities caring for patients who are ready for discharge to the community. Broadening these considerations to other populations of persons with chronic conditions we can summarize the critical success factors as follows. The first speaks to integrating services, and the second to identifying and addressing the needs of these largely neglected populations in policy and priority-setting work:

*1. Strong alliances built across ministry boundaries*

*Protocols developed for collaborative long-term planning, priority setting and funding within and across ministries using key stakeholder groups to help identify a publicly funded set of core programs and services for children, youth and adults with disabilities and chronic illnesses. Particular populations to consider include seniors (especially women), new and emerging populations living with disabilities and chronic illnesses, and persons with mental health and complex emotional needs*.

*2. Community health transitional priorities and benchmarks identified*

*An allied ministry strategy developed and implemented to identify transitional priorities and benchmarks for the core programs and services for children, youth, and adults with disabilities and chronic illnesses. Similar to initiatives to reduce acute health services wait times, community health transitional priorities and benchmarks include maximum wait times for community rehabilitation services, attendant care, and for supportive and accessible housing*.

#### Chronic illness and cancer care: services across the continuum

*Eric Thomas was 64 when he was diagnosed with prostate cancer and underwent a prostatectomy and subsequent chemotherapy and radiation. The treatment was successful and there had been no recurrence for the past 8 years. During his treatment, Eric was supported by an outreach cancer care nurse who provided education and information to help navigate the cancer care system. The local Community Care Access Centre provided a personal support worker (PSW) for two hours per week. These services were withdrawn when Eric went into remission and his active treatment ended*.

*Recently, Eric's prostate cancer was found to have reoccurred with metastases to his hip, lower back, and liver. A series of palliative chemotherapy and radiation treatments have been planned for what is now considered to be a terminal condition. Eric is now 72 years old and lives in a one-bedroom apartment with his wife, who has a heart condition and had cardiac bypass surgery only a few months ago. She is limited in her ability to assist Eric as his care needs increase*.

*Eric and his wife are concerned about his poor health and their limited resources to support him through this period. His pain is currently under control with Tylenol 3's, but he has great difficulty ambulating and keeping balance due to discomfort of movement. He is unable to get into the bathtub, and cannot stand for long periods. He sits in a reclining chair for most of eighteen hours of the day and is at risk for skin breakdown. Meal preparation is also challenging for the couple. Eric and his wife have no immediate family in close proximity and have a limited social circle of friends*.

#### LHINs principles

This is a difficult yet typical case encountered frequently in the community. Patients with chronic illnesses often do not receive appropriate follow up services and assistance either because of lack of availability or lack of awareness of these of these services [34]. Promoting the principle of *shared accountability *in particular would help address a number of the issues raised by this vignette. While responsibility and accountability for Eric's care was reasonably clear when he was under active treatment for cancer, accountability for his follow-up and the development of a longer-term care plan has not been clear, especially if he has not been referred to home care or other health and social services. Supportive and palliative services are not available in all communities, and many people do not know what options are available for themselves and their families. Even when patients are referred to one agency or another, accountability for their continuity and coordination of care and supportive services is unclear as they move back and forth between hospital and community sectors for treatment. In addition, active or palliative care scenarios aside, much of the everyday home care for people with chronic conditions (such as dressing, bathing, and feeding) is shouldered by the parents, spouses and offspring (most often women) of patients. These informal caregivers frequently have to juggle other responsibilities and may have their own health conditions that require attention [35]. This over-reliance on informal caregivers in such critical areas – a covert form of rationing – demonstrates the gaps in accountability for health care planning, coordination and delivery along the continuum of care and, thus, does not meet the principle of *shared accountability*. *Patient-centred care *is also at issue, as we read the principle broadly to include the needs of the patient's family.

What follows from the principle of *shared accountability *would be an approach to chronic illness management whereby a range of core supportive and palliative services are coordinated and easily available regardless of the patient's location – both home location, as well as location along the disease trajectory. Treatment centres and hospitals should ensure that their discharged patients and outpatients are aware of these services so that care levels are dictated by patient need rather than by timeline or treatment status. These services must be packaged around the needs of the individual patients and their families, with a focus on providing adequate pain control and symptom management, and appropriate caregiver supports [36]. Assistance must be given by discharge planners and case managers to patients and families in the development of a long-term care plan that addresses current and potential service needs, providing patients like Eric with choices in treatment options, knowledge of social and heath supports available (e.g., outreach nurses can often provide valuable assistance for patients with palliative and chronic needs) and access to information for informed decision making (including possible choice of place of death).

#### Critical success factor

Shared accountability thus is interpreted to include smooth transitioning for patients that are moving from one part of the health care "continuum" to another. This brings the care focus to the evolving needs of patients and their families rather than fragmented services offered by different facilities and agencies. Rather than having to navigate a complex care system, patients would have information and assistance in identifying needs and available services. This requires a patient-centred inter-sectorial decision making framework as per the following critical success factor:

3. *Accountability shared for flexibly tailoring resources and services to changing needs*

*A decision-making framework for shared accountability across health care and community sectors, agencies and ministries to ensure that core resources and services are not allocated piecemeal to individual patients and their families, but are strategically packaged and flexibly tailored to their changing needs*.

#### Senior's health: speech language pathology services

*Indira Prasad is a 72 year-old widowed woman who emigrated from India with her family in 1980. Indira's income is her basic pension (Old Age Security) and survivor's benefit (widow's allowance). She was never formally employed but worked to care for her family from the time she came to Canada. As she is widowed and her children are married, she now lives alone. Two years ago, Indira suffered a stroke and, after a brief hospitalisation, she was discharged home with a verbal speech deficit and some mobility difficulties that required that she walk with a cane to reduce the risk of falls. With a cane she is able to walk independently, however her inability to communicate verbally is impeding her day-to-day activities*.

*Access to speech language pathology (SLP) is very difficult to obtain in the community and waiting lists for outpatient services at rehabilitation hospitals are long. Past experience has shown that the only way to get timely SLP services is to pay privately. Indira does not have much money left after rent, utilities and food are paid to afford private SLP services. Her daughter has tried to advocate on her mother's behalf, but found the process frustrating and was unsuccessful in accessing treatment. As with many families that care for seniors with health problems, Indira's daughter feels the time and financial pressures of caring for her children and being a caregiver for her mother. Without access to timely services, Indira's quality of life and safety are compromised. With her language deficit, she is isolated and has difficulty doing daily activities such as banking and shopping. She is also at risk during an emergency*.

#### LHINs principles

The principle of *equitable access based on patient need *is key in this case and this principle is not met when only those with sufficient financial resources are able to access what could reasonably be considered a core service for people with disabilities and chronic illnesses. The many senior citizens without adequate finances to pay for expensive private services often face the prospect of nursing home placement sooner than desirable, which is more expensive than funding outpatient services and personal assistance programs for seniors living in the community. This case highlights how social disadvantage is often multiplied through the intersection of physical impairment with other factors like gender, age, ethnocultural identity, and socio-economic standing [37]. For example, gender is an important consideration when promoting seniors' *equitable access based on patient need *since senior citizen populations are disproportionately female and considerably more women than men live near or below the poverty line (at any age) [38]. This means that senior women are disproportionately disadvantaged by inability to pay for needed services like SLP, which are necessary to enable communication and, thus, meaningful community participation.

Furthermore, the principle of *responsiveness to local population health needs *is not met when seniors requiring outpatient rehab services are discharged despite the common knowledge that they will likely not be able to access those services in a timely manner. The alternative of extending their hospital stay in order to get those services, however in most cases would contradict the principle of *preserving patient choice *and *patient-centred care*, given that living independently at home is the goal of many seniors [39].

For a senior like Indira, functional needs and quality of life related to living at home would require access to publicly-funded speech language pathology services, an occupational therapist to do a home safety assessment, and a physiotherapist to follow up on walking safety. While public funds are not unlimited, the LHINs principles – together with the general commitment to reinvest funds from institutional to community sectors – support provision of such core services which are essential to safety and community participation. This includes personal assistance programs that help with daily activities like shopping, banking, and meal preparation that may also mediate the caregiver burden placed on loved ones. In addition, related to the principle of *shared accountability*, it is more cost-effective to provide outpatient and home support for seniors than to have them in and out of hospital with avoidable injuries or illness incurred at home, or "failure to thrive" episodes [40].

#### Critical success factor

Upholding the principles is not possible without taking seriously the numerous rehabilitation services that are under-funded or not funded because they are not considered "core". Many of these services are essential to the health and well-being of seniors and allow them to remain living safely in the community, prevent re-hospitalization and delay admission to long term care. The definition of what counts as a core service for these populations needs to be revisited and further investment considered as follows:

4. Differential impact of diversity and social position considered

*Expanded home care services for seniors, taking into account the differential impact of gender, age, ethnocultural identity, and socio-economic standing as part of a publicly funded set of core programs and services for people with disabilities and chronic illnesses who wish to continue living at home*.

### Community support services: new populations living with complex "paediatric" conditions into adulthood

*Mike Pritchard is a 21-year old man with Duchene Muscular Dystrophy who relies on nighttime ventilation, uses an electric wheelchair, and receives intensive human and technical assistance for most of his activities of daily living, including transfers, dressing, meal preparation, suctioning, and bladder/bowel care. Mike lives with his chronically ill mother in a rented house. He receives 1.5 hours of home care every morning, 5 days per week. His mother provides all the rest of his care throughout the day and on weekends. Their only source of income is social assistance*.

*Two years ago, Mike was discharged from the paediatric outpatient clinic that he had frequented since age 5. This clinic had offered comprehensive care, including bi-yearly multidisciplinary assessment, advice and care that included orthopaedic services, respiratory care, physical and occupational therapy, orthotics and seating. No similarly coordinated care services are currently available to him as an adult, and so he attends one clinic for his seating needs, another for his respiratory assessment, and another for cardiac monitoring. Attending these appointments requires a great deal of planning and coordination between his home care provider, transportation services, and the clinic. In addition, as Mike's condition progresses he encounters new health problems, most recently digestion difficulties, that few health care practitioners have the knowledge or experience to assess and treat*.

#### LHINs principles

The case of Mike Pritchard illustrates the shortage of needed services – and the inadequacy of their coordination – to meet the needs of such "new populations" (e.g., community ventilator-users, transplant recipients, cancer survivors, adults surviving complex conditions of childhood). The number of hours of home care that Mike receives does not meet his or his mother's needs, nor is access equitable given that comparable patients receive up to six hours of assistance per day [31].

The principle of *equitable access based on patient need *is not met when new populations do not have available, coordinated and integrated expert care. The principle of *shared accountability*, however, is likely most relevant to the problems surrounding treatment and services for new populations: there is clearly a need for "one-stop" core ambulatory programs and services provided by a range of professionals as per the coordinated, integrated paediatric clinic model. The convergence of services into an ambulatory clinic would also permit the health care professionals assembled to develop expertise, improve their practice, and conduct relevant research.

Adhering to the principles governing the LHIN model suggests the need for properly funded, multidisciplinary ambulatory services for adults with complex paediatric illnesses as well as programs to transition youths from paediatric to adult services. Furthermore, professional expertise with respect to the unique healthcare needs of these and other "new" populations must be developed.

#### Critical success factors

This vignette highlights the need for decision-makers to consider emerging "new" populations of health care recipients who have long-term care needs in applying the guiding principles to planning and program development. The needs of this relatively new and growing cohort of health care recipients have not been comprehensively identified. As we suggested in the first vignette, "equitable access based on patient need" can only be addressed once needs are known. What *is *known is that many of these individuals have complex chronic health and social service requirements that the current system does not support in part because of lack of capacity and expertise. In addition the complexity and multiplicity of the needs of new populations require that different parts of the health and social service system work in concert to share accountability and deliver quality care.

Our critical success factors thus encompass three areas – identifying needs, building capacity and integrating services:

**5. *Needs of new populations identified***. *Publicly funded subset of core programs and services most needed by new populations of people with disabilities and chronic illnesses identified*.

***6. Service providers trained to meet the complex needs of new populations with disabilities and chronic illnesses***. *A human resource strategy, including interprofessional and interdisciplinary curricula, developed and implemented to address the shortage of service providers for new populations of children, youth and adults with disabilities and chronic illnesses*.

**7. *Increased capacity and flexibility to address care needs of new populations***. *An allied ministry strategy developed and implemented with shared accountability toward providing new and emerging populations with disabilities and chronic illnesses with the coordinated, integrated, and patient-centred care they need, including rehabilitation, complex continuing care, mental health and emotional support*.

### Children's health: demands on parents to advocate for and coordinate care

*Logan Devonshire is a 5 year-old boy who was recently diagnosed with an Autism Spectrum Disorder. Along with difficulties socialising with other children and maintaining attention, Logan exhibits self-injurious behaviours that require him to be constantly supervised. Logan's challenges place a considerable strain on his parents and siblings. His family has applied and received funding from the Ministry of Community and Social Services' Special Services at Home (SSAH) program. Unfortunately, the amount they receive changes yearly according to the total funds available to the Ministry and the family is not eligible for a case manager. As a result, the Devonshires find it very difficult to navigate and coordinate a complex set of medical, social, and education services and supports across numerous agencies and organisations. Despite the parents' best efforts, there are intermittent gaps in Logan's care plan as a result. Furthermore, these efforts take up their time and take them away from professional and personal responsibilities. The family also has financial difficulties, as Mrs. Devonshire had to quit her job in order to devote time to Logan's care *[41].

#### LHINs principles

The complexity involved in accessing supports and different funding sources – and the position that parents are placed in where they have to "work" the system to meet the many needs of children with disabilities – shows that the principle of *shared accountability *has not been met. Parents find their advocacy and coordination roles challenging in part because they have to access numerous ministries (Health, Education, Children and Youth Services, Community and Social Services) in order to receive the available care. Without a case manager who is knowledgeable about the complex system of agencies, ministries, and service providers, parents must negotiate their children's care on their own. *Patient-centred care *would suggest services be organised around the needs of the child and family, including ready coordination between ministries, community services, and public and private payers. Without such a system in place, the principle of *equitable access based on patient need *is not met because families are straining to support their children's basic needs. The burden is evident in research that shows the negative effect on employment for parents of children with disabilities [42].

The LHINs principles would seem to dictate that services for children with disabilities and their families should be better coordinated to reduce bureaucratic hurdles. Doing so would clarify lines of accountability and make it simpler for families to access needed services. It would also allow case managers to assess needs more holistically, reduce gaps in services, and take a *patient-centred care *approach. While parents and guardians are key participants in the establishment and implementation of care plans for these children, access should not depend on the tenacity of parents and guardians to advocate for the care that children require.

#### Critical success factor

The applicable principles in this vignette parallel those in the prostate cancer vignette ("Eric Thomas") discussed above but in relation to children with chronic conditions and their families. Problems of identifying and navigating services offered by health care, education and social services can be incredibly trying for parents, particularly mothers, who spend considerable energy and resources in these endeavours. Mothers commonly give up employment in order to coordinate and manage their child's health care. Thus we suggest a responsive system that honours the guiding principles would provide coordinated services and case management as follows:

***8. Coordinated system of care for children with disabilities and guidance for families***. *A coordinated system of care with transparent linkages between the ministries, services, and funding agencies that provide supports and care for children with disabilities and chronic illnesses, including the youth-to-adult transition. In particular, assign families of such children a case manager with the authority to develop and implement a comprehensive patient-centred care plan, the ability to commit resources across funding envelopes, and the mandate to assist families in "navigating" the continuum of care*.

### Health promotion: diabetic education and community health

*Paula Arbour is a 55-year old woman with diabetes living in a rural First Nations community with a stressful job and many pressures on her time. Paula maintains a poor diet, rarely participates in recreational activities, and has put on excessive weight. She also has a family history of diabetes. With diabetes, particularly if it is not well managed, Paula is at risk of stroke, visual impairment, and diminished kidney function that could lead to the need for dialysis. Paula is worried about these risk factors and has expressed interest in learning strategies to minimize them; however there are no diabetic education programs available in her community*.

#### LHINs principles

Given that health promotion through education is most effective when tailored to the needs of affected communities, the most applicable principle in this case would appear to be *responsiveness to local population health needs*. For example, with timely access to diabetic education and health promotion, Paula could avoid harm to herself, stress to her family, and costs to the community by managing and improving her condition before the onset of serious illness. As diabetes is a growing problem, particularly within certain communities such as First Nations, this principle would suggest the expansion of accessible diabetic education programs tailored to the needs of the relevant communities.

Presently, most diabetic education is done in hospitals through outpatient programs that usually have lengthy waiting lists [43]. Priority is given based on the type and severity of diabetes. The programs offer counselling from dieticians and nurses, and social workers assist with stress management. However, since the health of people with diabetes may be compromised by the long waiting times, hospitals tend to rely on Community Care Access Centres support to provide education until space opens up in more comprehensive hospital diabetic education centres. In rural locations, such services are often not available.

The major shortcoming of these diabetic education centres is lack of accessibility, particularly due to lengthy waiting lists, inflexible hours to accommodate people's often busy and stressful schedules, and lack of rural programs. This challenge also calls into question whether the principle *equitable access based on patient need *is being satisfied. Furthermore, flexible hours would *preserve patient choice *and enhance *patient-centred care*. Continued research to assess growth trends in diabetes, assessing capacity to respond to demand and where best to provide diabetic education centres would be an element of *shared accountability*.

Following on these principles, community health promotion initiatives and health education centres should be put in place to help individuals manage their health and increase the health of communities as a whole. For instance, accessible diabetic education centres with flexible hours accommodating people's work schedules would help people like Paula improve their well-being through education and follow up regarding diet, stress management, and exercise. People diagnosed with chronic illnesses need timely access to information and early intervention strategies to avoid acute health problems and better manage the stresses of illness and disability. In rural areas, where such centres are not available, community health nurses and other health professionals should be properly trained in diabetic education. Counselling and follow up could reduce the risk for the onset of other illnesses [44]. Timely health promotion and funded outpatient activities, such as supervised exercise programs for at-risk groups, can improve health outcomes and provide other social benefits. These benefits include less time off work due to illness, decreased caregiver burden if an illness occurs, greater productivity, less cost in health care services and improved quality of life.

#### Critical success factor

Despite wide recognition that health promotion has the potential to improve the health of populations and reduce health expenditures, program availability remains sporadic and concentrated in urban centres. This critical success factor highlights the need to address discrepancies in rural and urban services in accordance with the principle of equitable access based on patient need especially given the unique needs and challenges facing urban versus rural regions:

***9. Innovative, flexible, capacity-building health promotion and education programs***. *Innovative, flexible, capacity-building health promotion and education programs developed in a widely accessible range of formats to meet the needs of diverse urban and rural communities in a timely way, focusing on client and professional education related to self-management of disabilities and chronic conditions*.

### Mental health and addiction services: meeting patients' complex physical and mental health needs

*Melissa Wang is a 43 year-old long-standing patient in a complex continuing care facility. She has several medical conditions, including Primary-Progressive Multiple Sclerosis (with behavioural and emotional sequelae) and osteoporosis, and has also been diagnosed as having Borderline Personality Disorder. She is without family or friends. Melissa is dependent on staff for all her care needs. Very few staff members in the complex continuing care facility, however, have any training in the area of mental health even though these skills are increasingly required. Melissa's mental health needs and the staff's inability to address them make providing for even her basic physical needs very challenging. Melissa is also verbally and physically abusive to staff. She feels staff treat her punitively, wants a new attending physician, and states that she is not happy at this facility*.

*Nursing staff report feeling overwhelmed – there is increased sick leave on the unit, staff are presenting to occupational health with stress issues, and some have left the unit citing the challenges caring for this patient as the primary reason. None of the physicians have been able to maintain a therapeutic relationship with the patient, and her current physician is the last available option. Melissa's health care team has arranged for all available mental health supports (such as a mental health nurse, psychologists and psychiatrists from a partner institution, liaison and outreach services) but these are sporadically available and insufficient*.

#### LHINs principles

Certainly all five of the LHINs principles are relevant to this difficult case at the intersection of complex continuing care and mental health, yet the key principle appears to be *equitable access based on patient need*. Due to the high level of specialty care required by patients with significant physical disabilities, these patients cannot access adequate mental health services when needed. Instead, such patients must rely on preliminary assessment of mental health problems by staff that often have inadequate training and must receive treatment via out-patient and out-reach services, which generally are inadequate to meet their mental health and complex emotional needs. Care delivery models are such that patients can receive either in-patient care for their physical health needs or in-patient care for their mental health needs, but not both. In instances where the level of physical care need is high, these needs are seen as primary. In such cases, patients cannot be said to have *equitable access *to the mental health services they need. Moreover, the health system's response to such patients is neither *patient-centred *nor *responsive to local population health needs*. Nor, under these circumstances, is there is a meaningful range of options sufficient to *preserve patient choice*.

If the principles, particularly *equitable access based on patient need*, were upheld in this context, we would expect to see an increase in the range of patient-centred, mental health services offered along a full continuum of care, as well as health care professionals trained to care more holistically for patients with physical disabilities, including their complex emotional, behavioural and/or mental health needs.

#### Critical success factor

This vignette highlights common problems encountered when differentiated care organizations are not oriented to caring for patients with complex needs that cross over the different "silos" of the health care system. Providing care for patients with both physical and mental health needs provides a particular challenge to honouring the principle of equitable access based on patient need. Our final critical success factor calls for improved capacity in this neglected area of care:

**10. *Services that meet the mental health and emotional care needs of people with disabilities and chronic illnesses implemented***. *Publicly funded subset of core mental health and emotional care programs and services most needed by people with disabilities and chronic illnesses identified and a human resource strategy (including interprofessional and interdisciplinary curricula) developed and implemented to address the shortage of service providers for children, youth, and adults with disabilities and chronic illnesses with a specialisation in mental health and complex emotional care*.

## Summary

In this paper we have used the example of Ontario to explicate guiding principles and derive critical success factors that can be employed by decision makers in examining the ethics of local regionalized health care practices. Using a series of vignettes that illustrate common ethical issues in rehabilitation and community care, we identified important integration opportunities that the LHINs model has the potential to address. We envision the critical success factors being used by LHINs decision-makers to guide the development and evaluation of Ontario's new regionalisation strategy.

Towards this end we have shared our analysis with the MOHLTC and LHINs leadership and collaborated on a recent MOHLTC "think tank" on ethical decision-making and priority setting for LHINs [45]. Our next step is to work with our Joint Centre for Bioethics priority setting group to serve as a resource for the LHINs leadership in developing an ethical framework for priority setting decisions. This work will include further clarification and specification of the LHINs principles and identifying other principles that might be relevant to priority setting processes. For example what specifically a principle of "equitable access based on patient need" demands of decision-makers in their local contexts and what others principles and values are necessary considerations in decision-making. If the MOHLTC moves forward with an explicit ethical framework for LHINs regionalized decision-making, to the best of our knowledge it would be the first Ministry of Health in Canada to do so.

This paper has integrated health services research, disability studies and ethics to identify and address a significant gap in the literature. Our analysis was limited by its focus on Ontario and its inheritance of the MOHLTC mid-level principles and did not tackle the important issues of priority setting which we view as an obvious next step. We nevertheless suggest the identified issues and principles provide a necessary foundation for further work and have wide applicability across jurisdictions to the provision of care and service to people with disabilities and chronic illnesses. Furthermore the critical success factors are broadly-based enough that they can serve as a normative point of departure for development and evaluation of regionalized systems that can be further specified to local contexts. In order to determine how these critical success factors could be met, targeted research in resource allocation and priority setting is a necessary complement. In addition, a fully articulated social justice framework that takes account of the needs of people with disabilities and chronic illnesses within health systems (regardless of delivery model) needs to be further developed.

### Summary of critical success factors

*1*. ***Strong alliances built across ministry boundaries*.**

Protocols developed for collaborative long-term planning, priority setting and funding within and across ministries using expert panels to help identify a publicly funded set of core programs and services for children, youth and adults with disabilities and chronic illnesses. Particular populations considered include seniors (especially women), new and emerging populations living with disabilities and chronic illnesses, and persons with mental health and complex emotional needs.

*2*. ***Community health transitional priorities and benchmarks identified*.**

An allied ministry strategy developed and implemented to identify transitional priorities and benchmarks for the core programs and services for children, youth and adults with disabilities and chronic illnesses. Similar to initiatives to reduce acute health services wait times, community health transitional priorities and benchmarks include maximum wait times for community rehabilitation services, for attendant care, and for supportive and accessible housing.

*3*. ***Accountability shared for flexibly tailoring resources and services to changing needs*.**

A decision-making framework developed for shared accountability across health care and community sectors, agencies and ministries to ensure that core resources and services are not allocated piecemeal to individual patients and their families, but are strategically packaged and flexibly tailored to their changing needs.

4. *Differential impact of diversity and social position considered*

Expanded home care services for seniors, taking into account the differential impact of gender, age, ethnocultural identity, and socio-economic standing as part of a publicly funded set of core programs and services for people with disabilities and chronic illnesses who wish to continue living at home.

**5. *Needs of new populations identified***.

Publicly funded subset of core programs and services most needed by new populations of people with disabilities and chronic illnesses identified.

**6**. ***Service providers trained to meet the complex needs of people with disabilities and chronic illnesses*. **A human resource strategy, including interprofessional and interdisciplinary curricula, developed and implemented to address the shortage of service providers for children, youth and adults with disabilities and chronic illnesses.

7. *Increased capacity and flexibility to address care needs of new populations*.

An allied ministry strategy developed and implemented with shared accountability toward providing new and emerging populations with disabilities and chronic illnesses with the coordinated, integrated, and patient-centred care they need, including rehabilitation, complex continuing care, mental health and emotional support.

**8. *Coordinated system of care for children with disabilities and guidance for families*. **A coordinated system of care with transparent linkages between the ministries, services, and funding agencies that provide supports and care for children with disabilities and chronic illnesses, including the youth-to-adult transition. In particular, families of such children assigned to case managers with the authority to develop and implement a comprehensive patient-centred care plan, the ability to commit resources across funding envelopes, and the mandate to assist families in "navigating" the continuum of care.

**9**. ***Innovative, flexible, capacity-building health promotion and education programs developed***. Innovative, flexible, capacity-building health promotion and education programs developed in a widely accessible range of formats to meet the needs of diverse urban and rural communities in a timely way, focusing on client and professional education related to self-management of disabilities and chronic conditions.

**10**. ***Services that meet the mental health and emotional care needs of people with disabilities and chronic illnesses implemented***. Publicly funded subset of core mental health and emotional care programs and services most needed by people with disabilities and chronic illnesses identified and a human resource strategy (including interprofessional and interdisciplinary curricula) developed and implemented to address the shortage of service providers for children, youth and adults with disabilities and chronic illnesses with a specialization in mental health and complex emotional care.

## Competing interests

The author(s) declare that they have no competing interests.

## Authors' contributions

Barbara Secker was responsible for the overall development and analyses, conducted and analysed the Delphi process, and was primary author of two of the vignettes

Maya J Goldenberg executed and coordinated the writing of drafts of the paper and contributed to the analysis

Barbara E Gibson contributed to the analysis, was primary author of one of the vignettes and primary author of the methods section.

Frank Wagner contributed to the analysis, was primary author of one of the vignettes and assisted with the Delphi process

Bob Parke contributed to the analysis and was primary author of two of the vignettes

Jonathan Breslin and Alison Thompson contributed to the analysis

Jonathan R Lear conducted the review and summary of the literature and contributed to the analysis

Peter A Singer contributed to overall conceptualization of the purpose, methods and deliverables of the study and contributed to the analysis

## Pre-publication history

The pre-publication history for this paper can be accessed here:


